# Quality Assessment of the Neural Algorithms on the Example of EIT-UST Hybrid Tomography

**DOI:** 10.3390/s20113324

**Published:** 2020-06-11

**Authors:** Grzegorz Kłosowski, Tomasz Rymarczyk, Tomasz Cieplak, Konrad Niderla, Łukasz Skowron

**Affiliations:** 1 Faculty of Management, Lublin University of Technology, 20–618 Lublin, Poland; t.cieplak@pollub.pl (T.C.); l.skowron@pollub.pl (Ł.S.); 2 University of Economics and Innovation in Lublin Research & Development Centre Netrix S.A., 20-209 Lublin, Poland; tomasz@rymarczyk.com (T.R.); konrad.niderla@netrix.com.pl (K.N.); 3 Research & Development Centre Netrix S.A., 20-704 Lublin, Poland

**Keywords:** industrial tomography, machine learning, neural networks, cyber-physical system, hybrid systems, production process management, quality assessment

## Abstract

The paper presents the results of research on the hybrid industrial tomograph electrical impedance tomography (EIT) and ultrasonic tomography (UST) (EIT-UST), operating on the basis of electrical and ultrasonic data. The emphasis of the research was placed on the algorithmic domain. However, it should be emphasized that all hardware components of the hybrid tomograph, including electronics, sensors and transducers, have been designed and mostly made in the Netrix S.A. laboratory. The test object was a tank filled with water with several dozen percent concentration. As part of the study, the original multiple neural networks system was trained, the characteristic feature of which is the generation of each of the individual pixels of the tomographic image, using an independent artificial neural network (ANN), with the input vector for all ANNs being the same. Despite the same measurement vector, each of the ANNs generates its own independent output value for a given tomogram pixel, because, during training, the networks get their respective weights and biases. During the tests, the results of three tomographic methods were compared: EIT, UST and EIT-UST hybrid. The results confirm that the use of heterogeneous tomographic systems (hybrids) increases the reliability of reconstruction in various measuring cases, which is used to solve quality problems in managing production processes.

## 1. Introduction

This article presents the results of research on the hybrid industrial tomograph electrical impedance tomography (EIT) and ultrasonic tomography (UST) (EIT-UST), operating based on electrical and ultrasonic data. A particular emphasis of the research was placed on the algorithmic sphere. However, it should be emphasized that all hardware components of the hybrid tomograph, including electronics, sensors and transducers, have been designed and mostly made in the Netrix S.A. laboratory. The test object was a plastic tank filled with tap water, inside which plastic tubes of different diameters filled with air were placed.

Industrial tomography, also called process tomography, is a non-invasive method of monitoring objects. This fact makes it finds increasing applications in various industries, such as food, chemical, cosmetics, pharmaceutical, metallurgical, waste utilization (biogas plants) and others. There are two main reasons for monitoring industrial processes. The first is to prevent equipment failures, which, in extreme cases, could result in production stoppage and even an ecological disaster (e.g., failure in the refinery system). The second key reason for monitoring industrial processes is the need for their optimization, which can take place on several levels. For example, optimization may involve minimizing production costs as a result of shortening the production cycle, reducing material costs or increasing the level of automation in controlling production processes [1].

Chemical processes inside reactors relate to phase transitions involving liquid, gas or solid phase. The monitoring of such processes mainly aims to tracking the spatiotemporal changes occurring in the contents of the tank. These changes may relate to the distribution and size of gas bubbles, forming crystals, the presence of impurities (inclusions) or local and global retention of the liquid phase. Techniques based on the correlation between hydrodynamic variables and mixing indices, as well as the interphase mass transfer coefficient, can be used to monitor and control the chemical processes occurring inside the reactor. Techniques such as residence time distribution and stimulus-responses are invasive, since they extract process characteristics from output to specific input disruptions. Consequently, they are limited to a narrow range of operational variables [2].

Currently, an increase in the popularity of non-invasive methods of monitoring industrial processes can be observed, which, combined with efficient and effective methods of data processing, are very attractive from the point of view of the company’s economics. Machine learning algorithms and artificial intelligence have a special position here [3,4].

### 1.1. Literature Review

Industrial tomography, due to its non-invasiveness, speed of operation, ease of automation, as well as the falling costs of implementing this technology, is an attractive solution to the problem of monitoring processes in real time. Tomography does not affect chemical reactions inside the tank. EIT sensors and electrodes, as well as active UST transducers, are located outside the test object or on its walls, and therefore do not interfere with the flow of liquid or change the physics of its movement. The use of a tomograph does not change the dynamics of chemical reactions occurring inside the tank reactor. Therefore, tomography can monitor both the flow of liquid in pipelines and the inside of tanks. Tomography uses many physical phenomena, so many types of this noninvasive method are used. The most popular tomographic methods are [1]: X-ray tomography [5], electrical capacitive tomography [6,7,8,9,10,11,12], electrical impedance tomography [13,14], magnetoacoustics tomography [15], ultrasound tomography [16], radio tomography [17] and hybrid methods, which are a combination of two and more methods [12,18,19].

Modern tomographic systems operate on the basis of carefully developed and tested algorithms. In addition to a reliable and efficient hardware layer, the software plays a key role. For obvious reasons, it is more difficult to replace the tomograph hardware than to upgrade the software. That is why in tomography an innovative approach to the IT and programmable sphere is extremely important. This is reflected in a large number of publications on new and improved algorithmic methods implemented in various types of tomography.

The essence of tomographic imaging is the need to solve the inverse problem. In addition, the inverse problem is often undetermined (fewer equations than unknowns). Due to the small number of electrodes or transducers, the number of measurements is small, less than the number of image pixels in which the voltage or other value is reproduced. This is the basic difficulty that determines so much work for many researchers and engineers. The inverse problem, otherwise known as ill-posed problem, can be effectively solved by using iterative computational techniques [20]. Due to the development of information technology, which causes a decrease in the cost of computing operations, machine learning is becoming increasingly popular. Currently, the most commonly used algorithmic methods can be divided into two groups. The first group consists of artificial intelligence methods, among which the most important position is occupied by various types of artificial neural networks. The second group consists of statistical methods such as least absolute shrinkage and selection operator (LASSO) and elastic net [21], least-angle regression (LARS) [22], k-nearest neighbors (KNN), naive Bayes, multivariate adaptive regression splines (MARS) [23], classification tree, support vector machine (SVM), gradient boosting machine (GBM) [18], principal component or partial least square regression [10]. There is a general trend that the importance of predictive algorithms is growing in industrial applications [24,25,26,27,28,29,30,31,32].

In addition to machine learning techniques, other approaches can be used for tomographic applications. An example is the Gauss-Newton method [33], which involves the use of sophisticated, adjustable models to describe the inverse problem using a heuristically defined predecessor [34]. Other examples of non-machine learning methods are the steepest gradient descent approach and Landweber iteration as a variant [35], the algebraic reconstruction technique [36], regularization using total variation [37], the split augmented Lagrangian shrinkage algorithm [38] or the method of generalized vector sampled pattern matching [39]. The above-mentioned methods have been known for several decades and currently constitute the canon of tomography. It seems that their potential has already been exhausted, which is why, along with the development of computer technologies, substantial progress in the field of tomography is made through the development of algorithms for data processing.

So far, many studies have been described, in which the authors propose the use of hybrid methods in electrical or ultrasound tomography. Hybridity is usually understood as a combination of different types of algorithmic methods. There are known scientific works, in which the connections of autoencoders with neural networks were used [40,41], LASSO or elastic net with ANN [21,42] or a combination of two different convolutional neural networks (CNN) [18,43]. This article describes a hybrid tomograph combining two tomographic methods—EIT and UST—in a single system. Similar attempts have already been successfully made, an example of which is the development of Yuanyuan Li et al. [44] on the detection of cancerous tumors in human tissues.

### 1.2. Goal and Novelties

The main purpose of this article is to evaluate the effectiveness of the EIT-UST hybrid tomograph, compared to non-hybrid EIT and UST tomographs, based on artificial neural network algorithms. The novelty presented in this article is the prototype EIT-UST hybrid tomograph dedicated to monitoring industrial tank reactors and pipes. In addition to the concept of connection and the use of tomographic data containing quite different physical quantities (electrical and time), a modified concept of multiple neural networks described in our previous publications was also used [16,45]. The authors’ own contribution included the design and development of a tomograph prototype in both the hardware and algorithmic layers.

**Figure 1:**
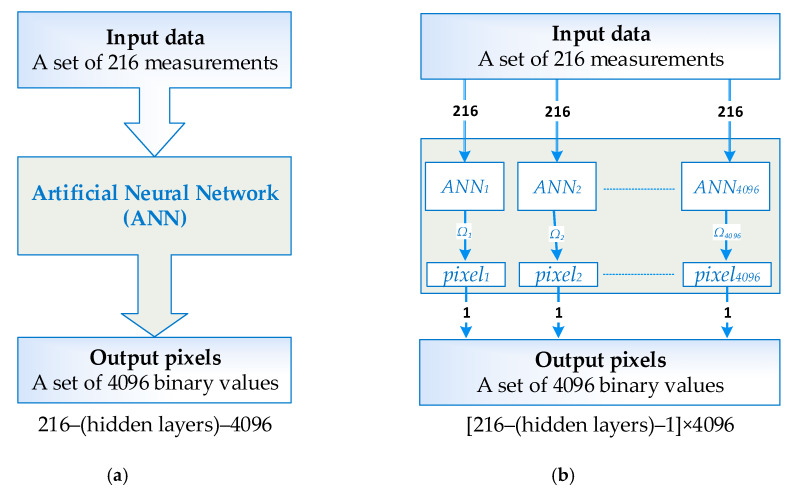
Comparison of algorithms for methods: (**a**) artificial neural network (ANN) and (**b**) multiple artificial neural network (MANN).

Figure 1 shows a comparison of a single artificial neural network (ANN) and multiple artificial neural networks (MANN). The measurement (input) vector contains 216 numerical values. The output image is created on a 4096-pixel grid. Hence, a typical ANN should have a structure of 216–(hidden layers)–4096. The MANN concept involves training separately as many single-output neural networks as the resolution of the tomogram (output image). Even though, in both ANN and MANN, the input vector is always the same, after training, the networks they differ in weights and biases. A single-output network is easier to train, which translates into better quality tomograms.

The article consists of four sections. Section 1 presents the theoretical aspects of industrial tomography and analysis of the literature in the area. Section 2 describes the key equipment components of the tomograph and how the algorithmic methods used. Section 3 presents the results obtained by using a hybrid tomograph. The results were compared with each other, in a way that allows verification of the effectiveness of the hybrid tomograph. The paper ends with Section 4, which contains our own observations obtained as a result of research and final conclusions.

## 2. Materials and Methods

### 2.1. Hardware

**Figure 2:**
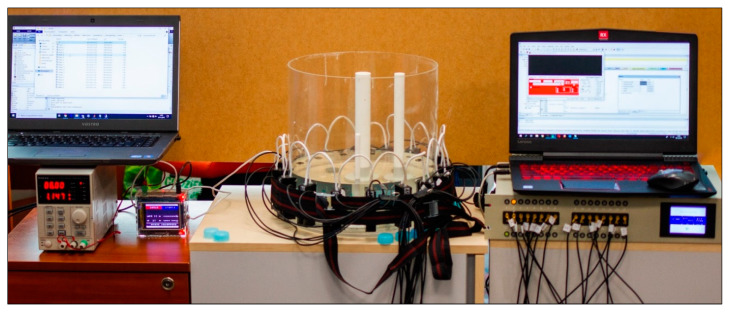
Test stand for experiments with the electrical impedance tomography (EIT) and ultrasonic tomography (UST) (EIT-UST) hybrid tomograph.

As described above, a prototype of a hybrid tomographic device was used during the study, enabling the creation of tomograms based on measurement data, derived simultaneously from electrical and ultrasonic measurements. To this end, our own electronic devices, electrodes and transducers, as well as algorithms, have been designed and developed. Figure 2 shows the test stand for experiments with the EIT-UST hybrid tomograph. In the center of the figure, a transparent tank surrounded by electrodes (EIT) and transducers (UST) is visible.

Around a tank with a diameter of 30 cm, 16 sensors were placed for each method—16 passive electrodes for EIT and 16 active transducers for UST. The design of the EIT electrodes is very simple. Their task is to generate and enable a reading of the potential difference between successive pairs of electrodes, switched utilizing a multiplexer.

**Figure 3:**
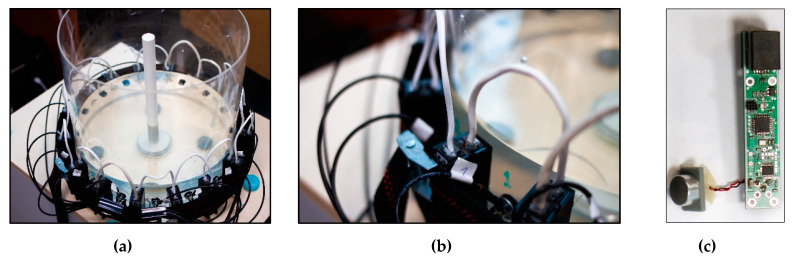
Research object: (**a**) tank with central inclusion, (**b**) connected electrodes and transducers, (**c**) inside the UST transducer.

Figure 3 shows the alternating arrangement of electrodes and transducers attached around the test tank. The fabric belt visible in the picture surrounding the system of electrodes and transducers is designed to provide a better pressure of these elements to the walls of the tank. The construction of an active UST transducer is relatively complicated, due to the need to simultaneously emit and receive ultrasound waves of a given frequency and amplitude. Therefore, RJ12 connection wiring was used to link, supply and communicate transducers with the tomograph. Each of the transducers has a built-in single-chip microcontroller with an A/C converter for processing audio signals, an active filter and a potentiometer to adjust the gain of received signals. The CAN 2.0A bus was used to read and control the measurements taken by the transducer. All transducers operate at 40 kHz.

Despite the fact that EIT electrodes and UST transducers are elements of one system, each set of EIT and UST sensors collects data independently of the other. In order to reduce interference and noise generated during measurements, the hybrid tomograph controls the moments of individual measurements, so as not to take electrical and ultrasonic measurements at the same time. More information on tomographic devices used in research can be found in our previous publications [16,46].

### 2.2. Electrical Impedance Tomography (EIT)

Electrical tomography involves solving the inverse problem, which can be determined by the relationship (1) [22]

(1) $$\nabla \cdot \left(\sigma \nabla u\right)=0\;\mathrm{for}\;\frac{\omega \varepsilon }{\sigma }\ll 1$$

where ∇u is the gradient of the potential distribution, *σ* is the electrical conductivity, *ε* is the permittivity, *ω* is the angular frequency.

**Figure 4:**
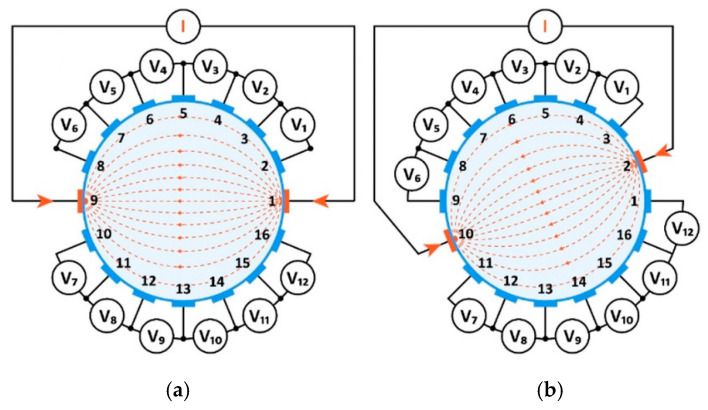
The opposite method of voltage measurement in a system of 16 electrodes [1]: (**a**) first measurement cycle, (**b**) next measurement cycle.

The arrangement of electrodes and the way of conducting measurements was analogous to that described in paper [1]. A total of 16 EIT electrodes were placed around the tested tank. An electric power source (I) was connected to a single pair of opposing electrodes. Then, between the adjacent electrodes, the voltage was measured as shown in Figure 4. As you can see, at a given projection angle, 12 voltage (V) measurements can be obtained from 16 electrodes. In Figure 4a, the projection angle is determined by the power source connected to electrodes 1 and 9. In Figure 4b, the projection angle is shifted, including electrodes 2 and 10. A single set of EIT measurements includes all projection angles, of which there are 8. After multiplying 12 measurements by 8 projection angles, we get 96 measurements, which is a single EIT measurement vector.

### 2.3. Ultrasonic Tomography (UST)

**Figure 5:**
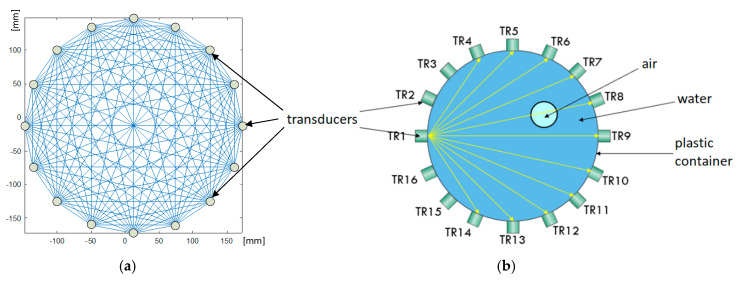
Principle of ultrasonic measurement using the transmission method: (**a**) the full grid of all UST signals, (**b**) signals emitted by a single transducer.

Figure 5 shows how the ultrasound measurements were performed. Due to the fact that each transducer can both emit and record sound waves, the so-called sound wave flight time between all transducers is collected in one series of measurements. The total number of all measurements for 16 transducers, with the proviso that the transducer does not receive its own signal is $16^{2}-16=240$. Assuming that the sound wave flight time does not depend on the direction of the sound wave propagation ($t_{1-2}=t_{2-1}$), the number of measurements can be reduced by half. In this case it is $(16^{2}-16)/2=120$. The way of calculating the number of UST measurements, depending on the number of transducers, was presented in the paper [16], and is determined by the Formula (2)

(2) $$M=\frac{n^{2}-n}{2}$$

where *n* is the number of transducers.

In Figure 5a, we see the density of the measurement grid against the background of the cross-section of the tested tank. The ultrasonic tomograph uses the phenomenon of varying propagation speed of a sound wave depending on the environment. Speed is correlated with time, so the UST system does not have to calculate the speed of wave propagation, but the time it needs to travel between individual transducers. Figure 5b shows the inclusion inside the tank through which the sound wave passes.

### 2.4. EIT-UST Hybrid Algorithm Principle

**Figure 6:**
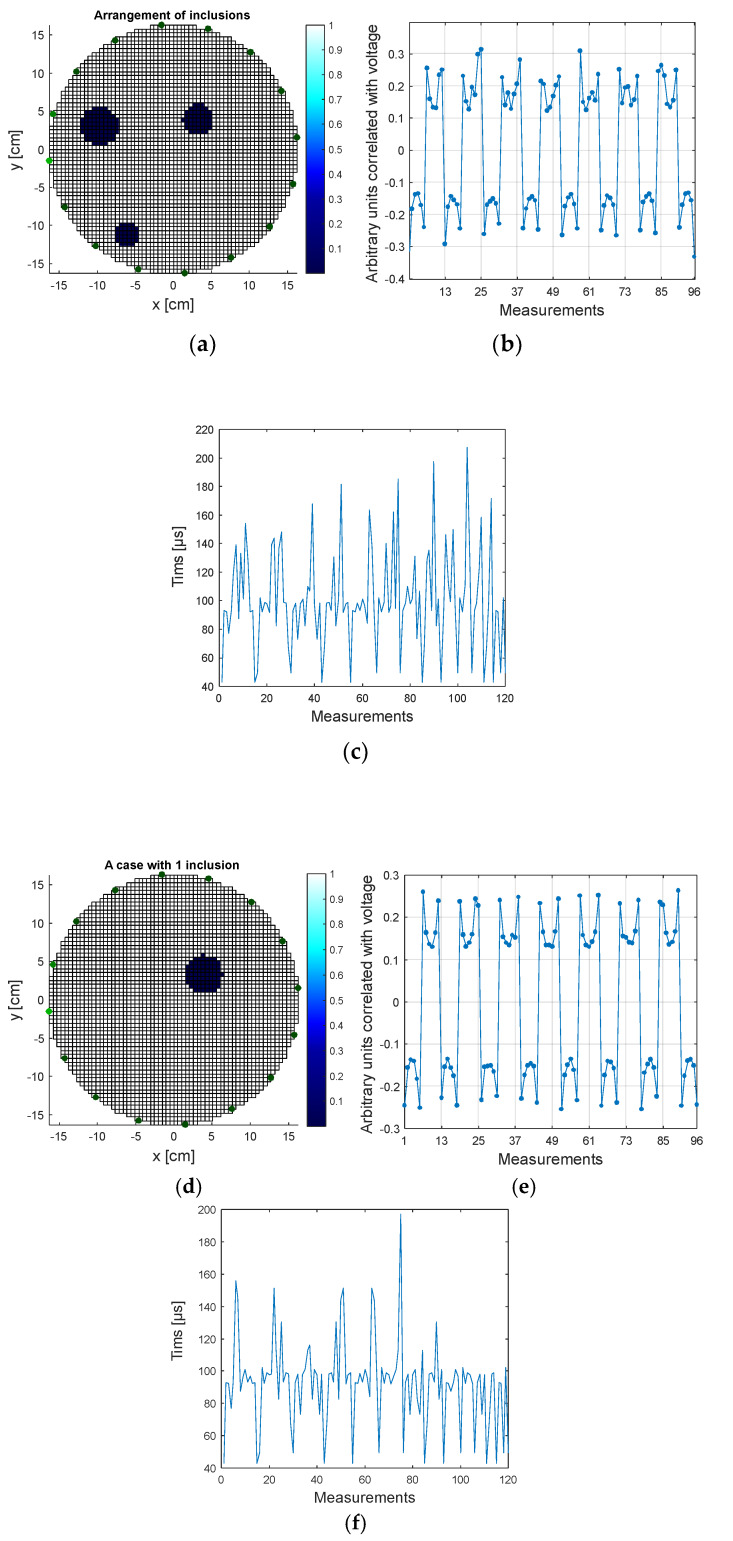
Real cases of measurement: (**a**,**d**) tomograms, (**b**,**e**) EIT arbitrary units correlated with voltage, (**c**,**f**) UST sound wave flight times between all transducers.

The developed prototype of the tomographic device is distinguished by the ability to record and process data coming simultaneously from electrodes (EIT) and transducers (UST). Based on the reference measurements, algorithms were developed to generate simulation data. The EIT measurement vector consists of 96 measurements, and the corresponding UST vector includes 120 measurements. Figure 6a presents 2 tomograms depicting real measurement cases. The first of them contains 3 inclusions, and the second one contains one inclusion. Figure 6b,c show the EIT and UST measurement values corresponding to the given tomograms. Unusual measuring units have been used for the EIT. Instead of directly measured voltage drops at individual electrode pairs, so-called arbitrary units that are correlated with voltage were used. Thanks to the use of arbitrary units, their values could be scaled, so that on the tomograms (Figure 6a), the white background consists of pixels with the value 1, while inclusions marked in dark color have the value 0.

Figure 6b,c show that the differences in EIT measurements for the two cases analyzed are more subtle than for UST. Thus, it can be concluded that for the examined environment the UST method can be used to obtain more satisfying results than the EIT method. However, because industrial tomography can be used in different conditions and on various objects, it cannot be assumed in advance which method (EIT or UST) will give better results. This kind of uncertainty justifies the use of hybrid tomography. Heterogeneous tomography is therefore more appropriate the greater the diversity and variability of the studied environment. The heterogenic type of tomography is also suitable for universal, portable commercial tomographs, which area of application should, by definition, be as wide as possible.

Two algorithms have been developed to generate simulation cases for UST and EIT. In order to increase the algorithm’s robustness, Gaussian noise has been implemented in the program code, with a separate level for each measurement in the frame. The noise level was determined by the standard deviation set to 5% of the value of each measurement. The UST case generating algorithm first creates a random, binary output image on a 64 × 64 square pixel grid. Then, to the randomly created tomogram, the algorithm matches 120 values of the measurement vector. In this case, the vector values are the sound wave flight times between transducers. The sound wave flight time evaluation algorithm is described in more detail in [16].

In the next step, the same reference tomogram serves as the basis for the second algorithm, whose task is to simulate the generation of 96 EIT electrical measurements. Finally, both UST and EIT vectors are merged into one vector with 216 (120 + 96) measurements.

**Figure 7:**
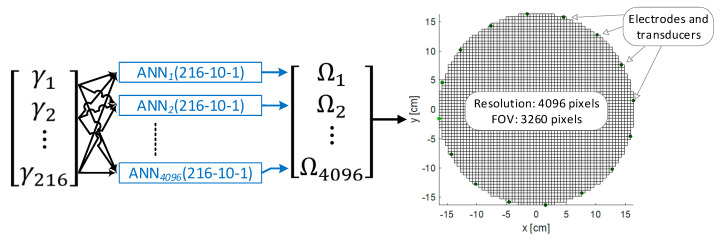
Multiple artificial neural network (MANN) system operation diagram.

Figure 7 shows a diagram of the multiple artificial neural network (MANN) system operation. At the input there is a measuring vector consisting of 216 values. The same vector feeds all 4096 ANNs, whose number corresponds to the number of pixels of the output image (tomogram). Each of the 4096 ANNs has a single Ω output value.

Each of the 4096 ANNs is separately trained to solve the regression problem. Despite the binary (categorical) nature of the output image, regression networks were used in the presented model. The reason is that if there is noise in the measurement data, binary images may contain many incorrect artifacts. They are therefore more difficult to interpret than regression images. Regression tomograms (multicolor), unlike monochrome ones, reproduce colors using real numbers. Thanks to this, pixels slightly different from the background reference value are hardly visible, while pixels with high deviations from the reference are more clearly visible. In the case of binary images, the key is to correctly parameterize the level of the cut-off filter, which decides which pixels will appear as inclusions and which will appear as the background. In the case of high noise level, this type of flirting is ineffective.

### 2.5. Neural Networks Training

In order to effectively implement MANN, the first step was to develop a set of data necessary to train neural networks. As previously mentioned, the hybrid data input vector includes 216 measurements. This number is the result of using 16 EIT electrodes and 16 UST transducers. A larger number of sensors (e.g., 32 for EIT and UST each) would require more space. This makes systems with more sensors more suitable for larger facilities.

#### 2.5.1. Data Preparation

**Figure 8:**
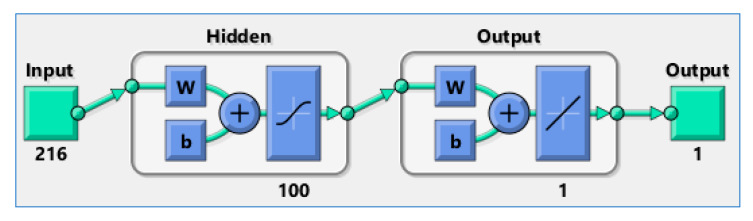
ANNs structure for EIT-UST hybrid data.

When designing neural networks, we always face the problem of selecting the size of a training set to ANN with a given structure. As a result of many attempts, and based on previous experience related to the use of neural networks in industrial tomography, it was decided that each ANN will have a single hidden layer containing 100 neurons. The number of 216 entries in the input layer was determined by the EIT and UST systems used. Assuming that each ANN generates the value of a single pixel, a tomographic output image with a resolution of 64 × 64, consisting of 4096 pixels determined the number of single ANNs needed to train. The structure of hybrid ANNs is as shown in Figure 8.

All ANNs operating within the MANN system have one hidden layer of 100 neurons. The number of neurons in the hidden layer was selected by the experimental method. Further increasing the number of neurons did not increase the effectiveness of prediction. All ANNs are feedforward networks with the tan-sigmoid transfer functions in the hidden layer and linear transfer function in the output layer. Each network has one output neuron, because there is only one target value associated with each input vector.

The number of hidden layers was not increased, because, according to the universal approximation theorem, a feed-forward network with a single hidden layer containing a finite number of neurons can approximate continuous functions on compact subsets of $R^{n}$, under mild assumptions of the activation function [47]. More than one hidden layer is usually used when there is information about the mathematical or physical significance of the underlying input-output relationship, or a single hidden layer would require estimating too many weights.

As previously described, measurements and corresponding images generated by measurement simulation algorithms were used to train the MANN neural predictor. The EIT measurement simulation uses the finite element method, based on the knowledge of the material parameter distribution (specific conductance). When developing the UST simulation algorithm, it was assumed that the sound waves in the tested tank propagate along straight lines. This avoids solving a complicated system of partial differential equations describing fluid mechanics.

Unfortunately, there is no simple relationship between the architecture of a multilayer network, and the minimum amount of training data that is necessary for the network to generalize responses and not fall into the trap of overfitting. Only an estimate of the upper and lower range of this number can be estimated. To determine the number of cases necessary to train neural networks with the assumed structure 216–100–1 (see Figure 8), Vapnik–Chervonenkis dimension (VCdim) Formula (3) was used.

(3) $$2\left[\frac{K}{2}\right]N\leq \mathrm{VCdim}\leq 2N_{w}\left(1+\log N_{n}\right)$$

where *K*—the number of neurons in the hidden layer; *N*—input vector dimension; $N_{w}$—total number of weights in ANN; $N_{n}$—total number of neurons in ANN. After calculating the different ANNs structures according to Formula (3), we obtain the results presented in Table 1.

As can be seen from Table 1, the use of 24,500 learning cases is a sufficient number to train ANNs with the structures listed in lines 1, 2, and 3. In addition to hybrid system (line 1), two homogeneous MANN systems—line 2 EIT and line 3 UST—were also trained. This was done with the intention of comparing the results of hybrid tomographic and homogeneous systems.

The last network in line 4 presents the hypothetical structure of a single ANN with 4096 neurons at the output, which could theoretically be trained to generate 64 × 64 resolution tomograms. According to the VCdim estimate for line 4, the minimum number of learning cases should be 884,736. Due to the very high level of network complexity, and the need to handle very large amounts of data, the effectiveness of learning such ANN is low, and the costs and time of training are high. This argument therefore suggests that simpler ANNs operating in parallel in the MANN system be used. The generated set of 35,000 cases was randomly divided in 70/15/15 proportions into learning, validation and test sets. This division is illustrated in Table 2.

#### 2.5.2. MANN Parameters and Training

The MANN structure described earlier includes 2 layers with transfer functions—a hidden layer and an output layer. All neurons in the hidden layer compute weights using the hyperbolic tangent sigmoid transfer function. The output layer uses the linear transfer function.

Training set covers as much as 70% of all cases, because its task is to provide basic information taken into account by transfer functions, based on which weights and bias values are determined during the ANNs training process. The cases belonging to testing set and validation set do not participate in the training process directly. In the ANN training algorithm used, the task of validation set was to prevent overfitting and thus to provide the network with generalization capability. Using an early stopping method, the network training algorithm calculated an error of the validation set [48]. If the validation error increases for the next six epochs, the network training process is interrupted.

Table 3 compares the learning outcomes of three types of neural networks: EIT, UST and the hybrid EIT-UST. The ANN quality was assessed on the basis of 2 metrics: mean square error (MSE) and regression (*R*). The comparison was made for a single, randomly selected pixel.

MSE error is calculated according to Formula (4)

(4) $$\mathrm{MSE}=\frac{1}{n}\overset{n}{\underset{i=1}{\sum }}{\left(\omega_{i}^{\prime }-\omega_{i}^{*}\right)}^{2}$$

where *n*—tomogram resolution; $\omega_{i}^{\prime }$—reference value of the pixel *i*; $\omega_{i}^{*}$—reconstruction value of the pixel *i*.

The formula for calculating the regression coefficient *R* is given by the Function (5):

(5) $$R\left(\omega^{\prime }-\omega^{*}\right)=\frac{cov\left(\omega^{\prime },\omega^{*}\right)}{\sigma_{\omega }^{\prime }\sigma_{\omega }^{*}}$$

where $\sigma_{y^{\prime }}$—standard deviation from reference values, $\sigma_{y^{*}}$—standard deviation of the reconstructed values.

Analyzing the metrics in Table 3, it can be seen that the smallest MSE and the largest regression *R* were obtained for ANN powered by heterogenous EIT-UST measurements. Slightly worse results were obtained for UST and the weakest for the EIT model.

**Figure 9:**
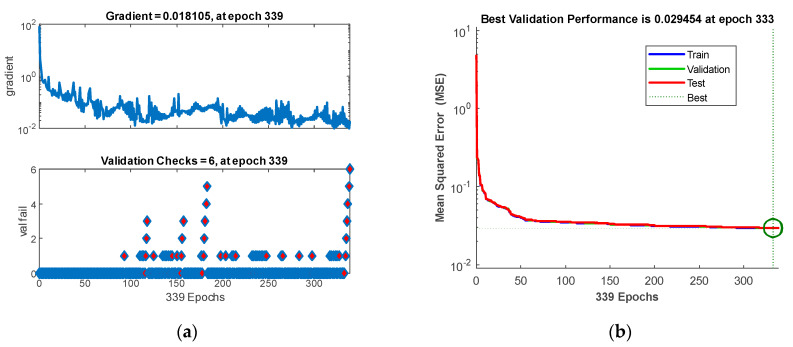
The training and validation process of the EIT-UST network: (**a**) early stopping method; (**b**) performance chart through the mean square error (MSE).

Figure 9a shows the gradient values for individual iterations of a validation set, in relation to the number of consecutive MSE increases for that set. This is important for the anti-overfitting method (early stopping), because after 6 consecutive increases of the MSE validation error, the ANN learning process is terminated.

Figure 9b shows the MSE values calculated for individual iterations for the training, validation and testing sets. As shown, all 3 lines are very concurrent and in shape resemble regular hyperbola, which indicates that the ANN is not overfitted. The best validation results were obtained at MSE = 0.0295, which is in accordance with Table 3.

**Figure 10:**
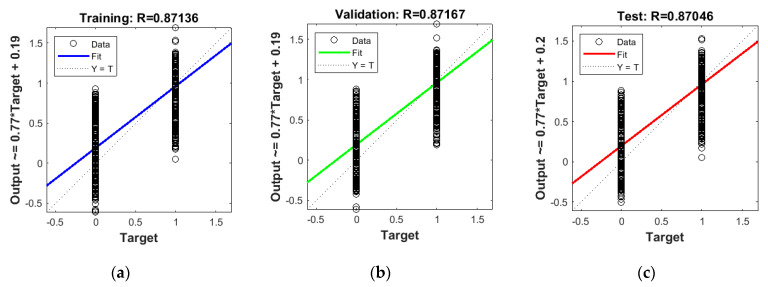
Regression statistics for (**a**) training set, (**b**) validation set and (**c**) testing set.

Figure 10 shows three R metric charts for three types of sets (training, validation and testing). The presented regression characteristics relate to the EIT-UST hybrid network and are consistent with Table 3. The vertical dispersion for two target values (0 and 1) is caused by the binary nature of the tomogram. Neural networks generate real (regression) values, so the number and spread of MSE errors does not necessarily indicate a low quality of prediction. MSE deviation values play a key role.

**Figure 11:**
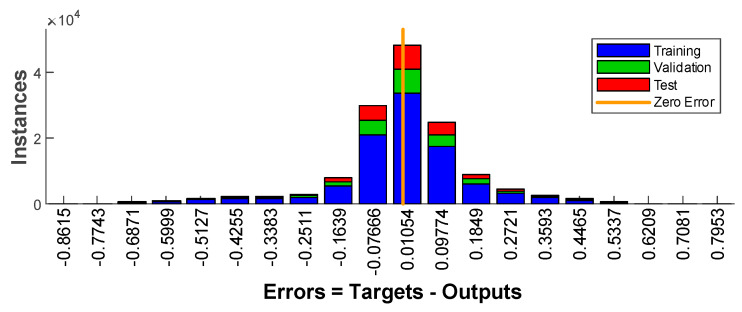
MSE histogram with 20 bins.

Figure 11 shows the MSE histogram for individual types of sets. It shows that by far the largest number of deviations has small values that are close to zero. It is a good indication of the quality of the neural network. It can be seen that the shape of the MSE histogram corresponds to the Gaussian curve, which also proves the correct ANN training.

## 3. Results

**Figure 12:**
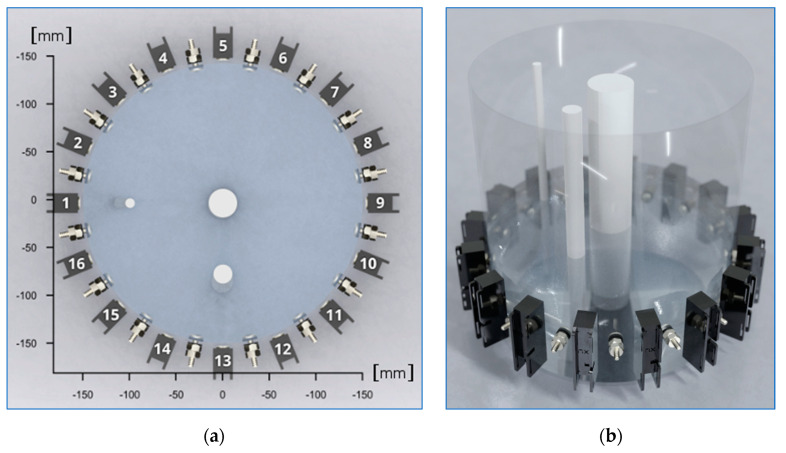
Model of the tested object with three inclusions: (**a**) 2D top view, (**b**) 3D side view.

This section presents the results of experiments performed on a real tank filled with tap water. The study used its own hybrid tomograph, which was designed and mostly made by its own research team. The data collected using the tomograph was processed using MANN algorithms. The results have been developed in a way that allows comparing the quality of operation of three tomographic methods: the EIT, UST and EIT-UST hybrid (heterogenous) method. Figure 12 shows the tank surrounded by EIT electrodes and UST transducers. Inside the tank, there are three tubes of different diameters filled with air.

### 3.1. Tomographic Reconstructions

**Figure 13:**
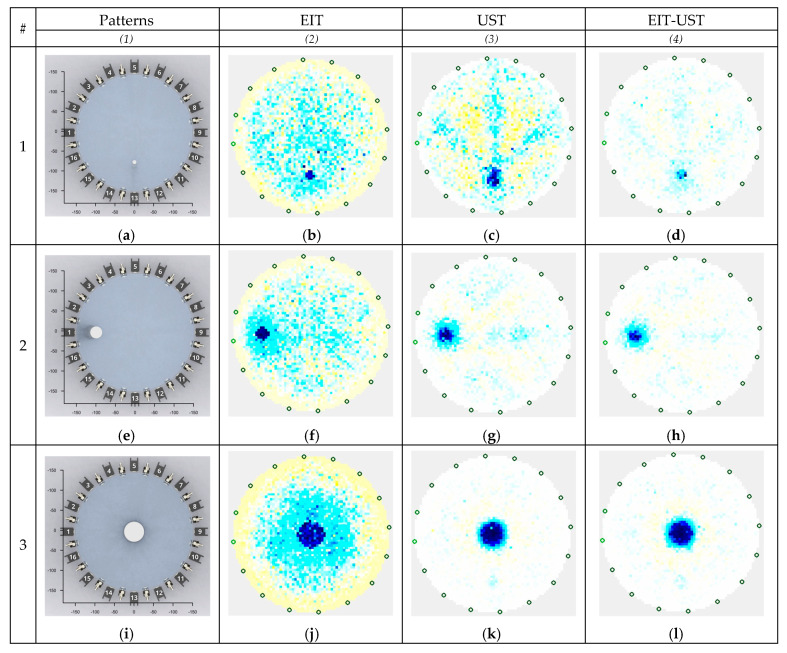

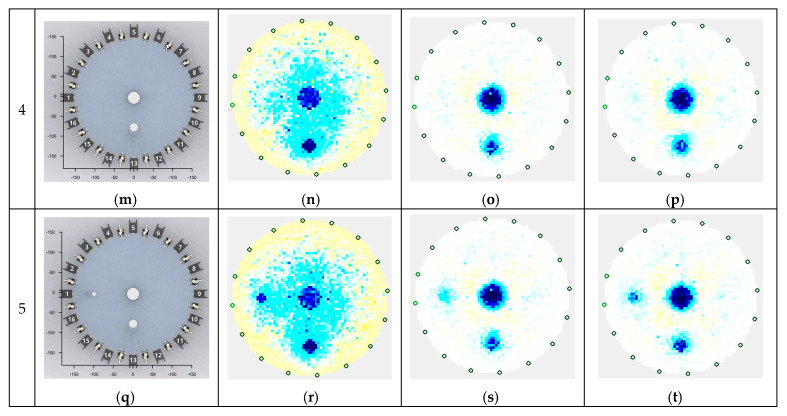
Reconstruction results based on real measurements: (**a**,**e**,**i**,**m**,**q**) – Patterns; (**b**,**f**,**j**,**n**,**r**) – EIT; (**c**,**g**,**k**,**o**,**s**) – UST; (**d**,**h**,**l**,**p**,**t**) – EIT-UST.

Figure 13 shows a set of tomograms obtained as a result of measurements. In the column (1) entitled "Patterns", there are reference images containing top views of the tested tank with spaced tubes. Around the water-filled tank, we see alternating EIT electrodes and UST transducers. It is worth paying attention to the dense packing of the sensors. Too small distances between the elements collecting measurement data may cause adverse effects in the form of interference and noise. Therefore, proper design of the measuring system has a great impact on the quality of reconstructed tomographic images.

The columns (2–4) contain the results of real reconstructions obtained with three methods: EIT (2), UST (3) and hybrid EIT-UST (4). Five cases differing in number, diameter and location of inclusions were compared. Individual cases are found in the lines numbered from #1 to #5 in Figure 13. Visual observation is a subjective method, and therefore it is not sufficient to compare the images fully objectively. However, it provides some insights that can be used for initial assessment. An ideal tomogram should have a white background and navy-blue inclusions. All other pixel colors with intermediate shades (yellow, gray, etc.) are noise and indicate mapping errors. It can be seen that tomographic images obtained with the EIT method appear to be noisier than UST and EIT-UST tomograms. However, this is not a rule, because the image (c) obtained by the UST method showing a small inclusion at the bottom of the tank also looks very noisy. In turn, all tomograms processed with the EIT-UST hybrid algorithm (d, h, l, p, t) are devoid of clear noise, although, in some cases, small inclusions can be hardly visible, as evidenced by the image (d). The general impression, however, is that the hybrid tomograph generates the most accurate images. To verify this hypothesis, an analysis based on objective criteria is needed. This type of analysis was carried out in the next subsection of this study.

### 3.2. Comparative Assessment of Methods and Algorithms

Three indicators were used to compare the three EIT, UST and EIT-UST tomographic methods: distribution error (DE), image correlation coefficient (ICC), and relative image error (RIE) [49]. The method of calculating DE is similar to MSE, but when comparing regression images, it takes the following form:

(6) $$\mathrm{DE}=\frac{1}{n}\overset{n}{\underset{i=1}{\sum }}\left|G_{i}^{\prime }-G_{i}^{*}\right|$$

where *n* is the number of pixels in the tomogram, $G_{i}^{\prime }$ is the reference value of the pixel *i*; $G_{i}^{*}$ is the reconstruction value of the pixel *i*.

The next metric is ICC, which can be calculated using the following Formula (7)

(7) $$\mathrm{ICC}=\frac{\sum_{i=1}^{n}\left(G_{i}^{*}-{\bar{G}}^{*}\right)\left(G_{i}^{\prime }-{\bar{G}}^{\prime }\right)}{\sqrt{\sum_{i=1}^{n}{\left(G_{i}^{*}-{\bar{G}}^{*}\right)}^{2}\sum_{i=1}^{n}{\left(G_{i}^{\prime }-{\bar{G}}^{\prime }\right)}^{2}}}$$

where ${\bar{G}}^{\prime }$ is the mean value for reference pixels and ${\bar{G}}^{*}$ is the mean value for reconstructed pixels.

RIE can be calculated according to Formula (8):

(8) $$\mathrm{RIE}=\frac{\left\|G^{\prime }-G^{*}\right\|}{\left\|G^{\prime }\right\|}$$

The best results from the MANN system should have minimum distribution error DE, minimum image error RIE and high correlation coefficient ICC.

Table 4 presents a comparative summary of the DE, ICC and RIE metrics for the three methods (EIT, UST and EIT-UST) and the five test cases numbered from #1 to #5. Table 4 is convenient for the vertical analysis of individual cases. In this way, the individual EIT, UST and EIT-UST methods can be compared for each of the five cases based on the DE, ICC and RIE criteria.

So, in each case and in the context of a given criterion, one of the three methods works best. The best values are underlined in Table 4. For example, for case #1, the best values of all metrics (DE = 52.97, ICC = 0.9949 and RIE = 0.0371) were obtained by the EIT-UST hybrid method, which is why these values were underlined. The last column, entitled "Mean", contains the arithmetic mean values of all five tested cases. It is a generalization that helps indicate the best method. As can be seen, the hybrid method prevailed in all three criteria.

Analyzing the results contained in Table 4, it can be seen that the DE error values are much higher than the other indicators. This is because the output image pixels have assigned one of two reference values—1600 (background value) and 330 (inclusion value). The ideal image should therefore consist of only pixels with the values of 1600 and 330. Since DE is an error mapping deviation, with such large output values, the example deviation DE = 52.97 (#1 for EIT-UST) should be treated as small. The assumed speed of the sound wave in water expressed in m/s is 1600, while 330 is the same speed in the air. The high value of the ICC correlation indicator is also noteworthy. In the literature, values exceeding 0.8 testify to a high level of image matching. To sum up, the analysis of evaluation metrics in Table 4 confirmed that the EIT-UST hybrid method outperforms the EIT and UST methods used independently.

## 4. Conclusions

The main purpose of the article was to verify the desirability of developing tomographic hybrid methods. A comparative assessment of three types of tomographs was carried out during the study: EIT, UST and EIT-UST hybrids. The obtained results make it possible to state unequivocally that the hybrid tomograph exceeded the quality of image reconstruction with other tested homogeneous methods (EIT and UST).

By analyzing the literature on tomographic methods and algorithms, as well as taking into account their own empirical observations, the authors of this publication came to the conclusion that an objective comparison of the tested method with the algorithms tested by other researchers would be possible, only if both the test object and the conditions in which studies were conducted were identical. For obvious reasons, meeting these conditions is extremely difficult. Therefore, the way the research was conducted was comparative, and the results obtained allowed us to verify the hypothesis about the higher effectiveness of the hybrid method over traditional methods.

The original approach to the algorithmic sphere deserves attention. Instead of a typical, single ANN network, the entire multiple ANNs (MANN) system was used, containing 4096 separately trained ANNs with a single neuron at the output. Each ANN supports a single tomogram pixel. Although this results in much more stringent requirements for MANN training, and for the reconstruction of tomographic images, the high quality of the images compensates for these inconveniences. The possibilities of training and using neural networks using efficient, multi-core CPU and GPU cause that the costs of obtaining high performance are quickly falling, and are proportional to the purchase prices of computer equipment. In the described studies, a PC equipped with an Intel Core i5 8400 CPU and NVIDIA GeForce RTX 2070 GPU was used to train the MANN systems.

The presented studies discussed in detail the comparison of EIT, UST and hybrid EIT-UST methods based on five cases. All cases concerned an identical test object—a tank filled with tap water—and were carried out under identical conditions (temperature, pressure, sensors, current-voltage parameters, etc.). Our empirical observations made during the testing of the hybrid tomograph on various objects, differing in the type of liquid (solution, suspension, etc.), its temperature, turbidity, fine particle contamination or flow rate, indicate that, in some circumstances, hybrid methods can be even worse than one of the homogeneous methods. Preliminary observations show that if the quality of the tomographic projections of the EIT and UST methods differ significantly from each other, then the combination of data (hybrid data) does not generate a synergy effect. In such situation, the measurements (input data) of one of the methods act as noise in relation to the measurements of the method more effective in the given conditions, thus deteriorating the result of the reconstruction. It seems that the synergistic effect of the hybrid tomograph is especially emphasized when separate, individual methods (e.g., EIT, UST), acting independently, give results of similar quality. The above aspects are the goal of further work on improving hybrid tomography conducted by our research team.

## Figures and Tables

**Table 1:** Vapnik–Chervonenkis dimensions for selected structures of ANNs.

#	ANN Structure	$\mathrm{VC}\dim_{min}$	$\mathrm{VC}\dim_{max}$	Number of Training Cases Used
1.	216–100–1	21,600	152,653	24,500
2.	96–45–1	4224	27,809	24,500
3.	120–55–1	6480	43,561	24,500
4.	120–4096–4096	884,736	174,040,058	not applied

**Table 2:** Division of data sets into training, test and validation.

All Cases	Training Set	Validation Set	Testing Set
100%	70%	15%	15%
35,000	24,500	5250	5250

**Table 3:** Division of data sets into training, validation and testing.

Set Type	EIT		UST		EIT-UST	
Evaluation metrics	MSE	*R*	MSE	*R*	MSE	*R*
Training set	0.0652	0.73093	0.0366	0.85855	0.0292	0.88855
Validation set	0.0644	0.72995	0.0370	0.85540	0.0295	0.88726
Testing set	0.0652	0.71986	0.0371	0.85404	0.0297	0.88653

**Table 4:** Comparison of image reconstruction indicators.

Evaluation Metrics	Methods	Tested Cases					Mean
		#1	#2	#3	#4	#5	
DE	EIT	68.94	81.69	160.45	155.46	167.25	126.76
	UST	64.22	69.73	74.14	83.67	100.34	78.42
	EIT-UST	52.97	65.27	73.65	81.84	79.16	70.58
ICC	EIT	0.9922	0.9886	0.9551	0.9578	0.9513	0.9690
	UST	0.9924	0.9911	0.9905	0.9879	0.9826	0.9889
	EIT-UST	0.9949	0.9922	0.9906	0.9884	0.9893	0.9911
RIE	EIT	0.0483	0.0574	0.1139	0.1103	0.1189	0.0898
	UST	0.0450	0.0490	0.0526	0.0594	0.0713	0.0555
	EIT-UST	0.0371	0.0459	0.0523	0.0581	0.0563	0.0499
